# Comparison of the Bond Strength of Silane-Treated Glass Fiber Posts at Different Solvent Evaporation Times: An In Vitro Study

**DOI:** 10.4317/jced.63939

**Published:** 2026-03-30

**Authors:** Valeria Ramos-Ramos, Sergio Dextre-Herrera, Giovanna Ramírez-Vargas, Leonor Castro-Ramirez, Denisse Turpo-Claudio, Luis Cervantes-Ganoza, César Cayo-Rojas

**Affiliations:** 1 School of Stomatology, Universidad Privada San Juan Bautista, Lima, Peru

## Abstract

**Background:**

Silane is used as a coupling agent on glass fiber posts to enhance chemical interaction at the post-resin cement interface. This in vitro study evaluated the bond strength of silane-treated glass fiber posts at different solvent evaporation times.

**Material and Methods:**

This in vitro experimental study included forty single-rooted mandibular premolars assigned to four groups according to silane solvent evaporation time: G1 (control), G2 (60 s), G3 (120 s), and G4 (180 s). Specimens were stored in physiological saline at 37 °C. After endodontic treatment, root canals were prepared and glass fiber posts were cemented. In the experimental groups, silane was applied and allowed to evaporate for the allotted time. After cementation, the roots were sectioned into thirds (coronal, middle, and apical) and bond strength was determined using the push-out test on a universal testing machine. Welch's ANOVA with Games-Howell post hoc was used to compare groups. Statistical significance was set at p &lt; 0.05.

**Results:**

In the middle third, significant differences were observed between evaporation times (p &lt; 0.001), with higher values for 180 s (13.94 ± 6.59 MPa) versus 60 s (3.86 ± 2.04 MPa; p = 0.004), 120 s (3.30 ± 1.85 MPa; p = 0.003) and control (4.69 ± 2.95 MPa; p = 0.007). In the coronal third (control: 7.09 ± 3.00 MPa; 60 s: 7.22 ± 3.16 MPa; 120 s: 4.81 ± 2.05 MPa; 180 s: 8.01 ± 4.34 MPa) and in the apical third (control: 6.49 ± 3.09 MPa; 60 s: 5.56 ± 1.96 MPa; 120 s: 5.19 ± 3.98 MPa; 180 s: 8.10 ± 3.40 MPa) no significant differences were detected between protocols (p = 0.164 and p = 0.196, respectively). When comparing root thirds within each protocol, only the 60 s group showed differences, with higher bond strength in the coronal third than in the middle third (p = 0.032).

**Conclusions:**

Evaporation of the silane solvent for 180 s improved the bond strength of the glass fiber posts in the middle root third, while no significant differences were detected in the coronal and apical thirds between the times evaluated. Within protocols, the 60 s group showed lower bond strength in the middle third than in the coronal third. Taken together, these findings suggest that prolonging evaporation to 180 s may be a useful operative adjustment to optimize bonding in the middle third, with no evidence of consistent benefit in the coronal and apical regions.

## Introduction

The rehabilitation of endodontically treated teeth with extensive loss of coronal structure remains a clinical challenge, as it requires restoring retention and function while ensuring a predictable long-term prognosis through reliable restorative strategies (1 , 2). In this context, intraradicular posts are indicated primarily to provide core retention and facilitate coronal reconstruction, rather than to reinforce the root (1 , 2). Synthesized evidence suggests that glass fiber and metal posts have comparable failure rates in endodontically treated teeth (3). Randomized clinical trials and systematic reviews with prolonged follow-ups have reported similar survival rates between fiber posts and cast metal posts, although clinically relevant differences exist in the type and manageability of some failures (4 - 6). Among the current alternatives, glass fiber posts have been consolidated for their compatibility with adhesive techniques and for their widespread use in esthetic and functional rehabilitations (7). However, even though overall survival may be comparable, loss of retention (adhesive decementation/debonding) is considered a critical and clinically relevant outcome, especially in teeth without a splinting effect (i.e., without a circumferential band of remaining coronal dentin) or with severe structural loss, where restorative stability is more dependent on intraradicular adhesive retention (4 - 7). The performance of fiber posts depends on the quality of the adhesive bond in the root canal, where unfavorable bonding conditions such as limited access, moisture control, high C-factor geometry, and restrictions for effective light-curing depth converge, favoring bond gradients and greater vulnerability toward the apical third (7 - 10). For this reason, bond strength is usually evaluated by means of the push-out test, since it allows estimating adhesive performance by root third and detecting differences in retention along the canal (9 , 10). Retention of fiber posts is modulated by variables such as resin cement type, adhesive strategy, application technique, and post surface pretreatment, with heterogeneous results among protocols and manufacturer recommendations (8 , 9). Consistently, it has been noted that the cement-post interface may be as determinant as the cement-dentin interface in explaining retention loss failures, which supports the interest in optimizing specific post conditioning variables (9 , 10). Glass fiber posts consist of fibers embedded in a resinous matrix (often epoxy), whose surface nature may limit direct chemical interaction with methacrylic cements, making the cementation process more dependent on well-controlled surface treatments (7 , 11). Therefore, mechanical or chemical pretreatments that increase surface energy and microretention have been proposed, often followed by silanization to promote chemical coupling between inorganic fibers and organic methacrylic matrices (11 , 12). However, the clinical and laboratory utility of silanization is not uniform, and its effect may depend on the cementation system and the complete conditioning protocol used (12 - 14). Silane agents are bifunctional molecules capable of linking silica-rich inorganic surfaces to methacrylic organic matrices through hydrolysis and condensation reactions, culminating in a siloxane network and copolymerization with resins (15 , 16). From a physicochemical perspective, the effectiveness of silanization does not depend solely on the mere application of silane, but on conditions that influence the formation of a stable film, including solvent type, the availability of water for hydrolysis, and, critically, the subsequent drying/solvent-evaporation step (15 , 16). Despite this, in vitro reviews and meta-analyses describe methodological heterogeneity and incomplete reporting on application parameters (including drying/evaporation), which limits standardization and may contribute to contradictory results and inconsistent clinical recommendations (8 , 14 , 15). Considering that bonding tends to be more vulnerable at depth, evaluating the effect of an operative parameter such as solvent evaporation time by root thirds provides information directly applicable to cementation in areas where retention is most critical (7 - 10). Identifying an evaporation time that optimizes bond strength could contribute to reducing decementation events and promoting more consistent and reproducible protocols for the cementation of glass fiber posts (4 - 8). In this context, the aim of the present study was to evaluate the effect of three solvent evaporation times after silane application (60, 120 and 180 seconds), compared to a control without silanization, on the push-out bond strength of glass fiber posts cemented in root dentin, analyzed by thirds (coronal, middle and apical). The null hypothesis was that there would be no significant differences in bond strength between the different silane solvent evaporation times and between the root thirds evaluated.

## Materials and Methods

**Figure 1:**
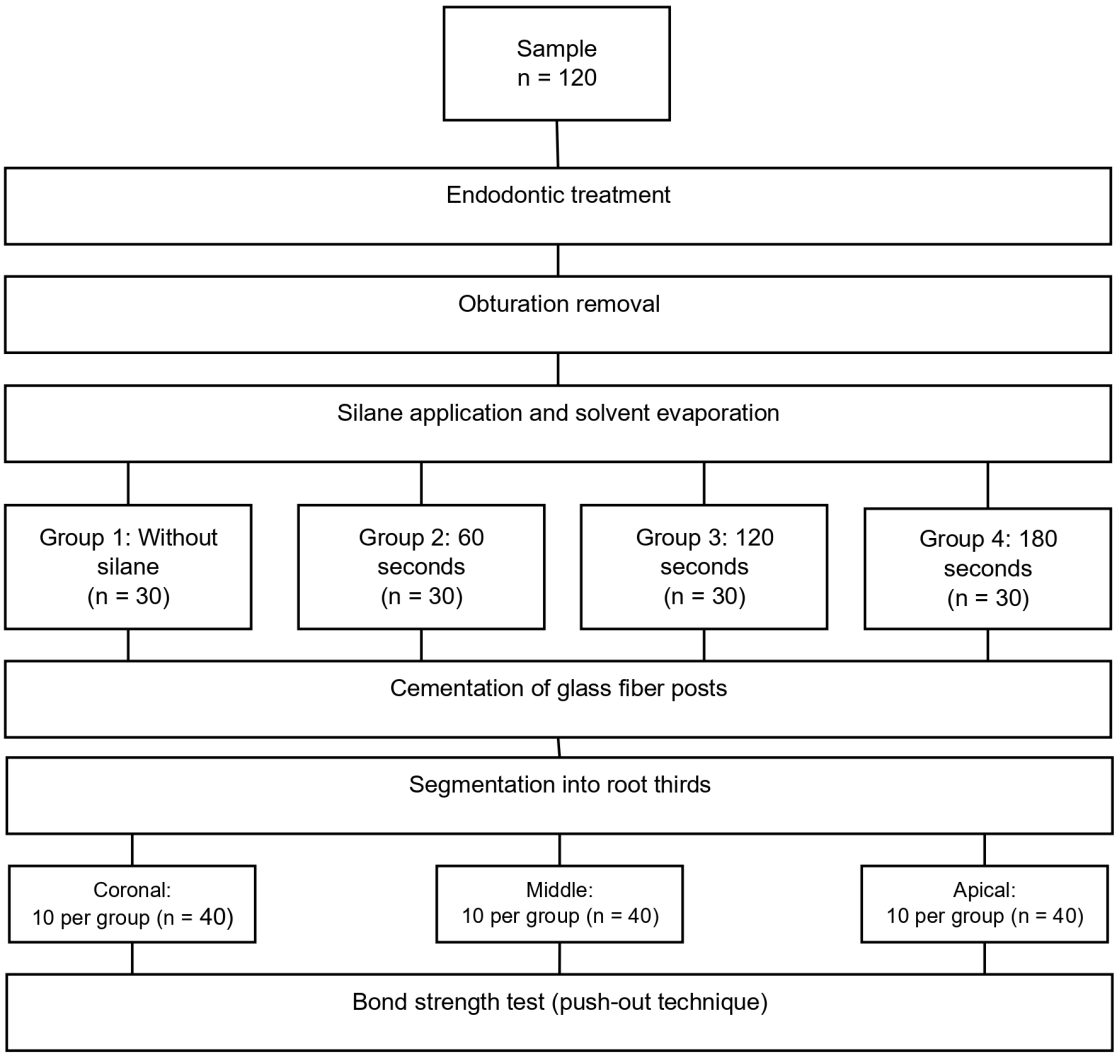
Random distribution of groups according to sample size.

1. Study design This in vitro experimental study was conducted at the School of Stomatology, Universidad Privada San Juan Bautista (UPSJB), and at an ISO/IEC 17025-accredited laboratory (High Technology Laboratory Certificate), Lima, Peru, between August and October 2025. The study protocol was approved by the Institutional Research Ethics Committee of Universidad Privada San Juan Bautista (Approval No. 898-2025-CIEI-UPSJB). The manuscript was prepared in accordance with the CRIS guidelines (Checklist for Reporting In-vitro Studies) (17). 2. Sample calculation and selection Forty single-rooted mandibular premolars extracted for orthodontic reasons were selected. Inclusion criteria were an intact external surface and the absence of caries, fractures, restorations, and discoloration. Teeth were stored in physiological saline at 37 °C until processing. Teeth with cracks, caries, restorations, or roots shorter than 15 mm (measured from the apex to the cementoenamel junction with a millimeter ruler) were excluded (18). Sample size was calculated using G*Power (version 3.1.9.7) in the analysis of variance family. The effect size (f = 0.957) was estimated from a pilot study (n = 5 per group). With = 0.05 and power (1) = 0.80, the minimum total sample size required was 36. To meet this requirement, 40 teeth were included. After endodontic procedures (root canal treatment and post-space preparation), teeth were randomly allocated to four groups according to silane solvent evaporation time: Group 1, control (no silane application) (n = 10 teeth); Group 2, 60 s (n = 10 teeth); Group 3, 120 s (n = 10 teeth); and Group 4, 180 s (n = 10 teeth). After post cementation, each root was divided into three thirds (coronal, middle, and apical) by two perpendicular cuts; from each third, one 1-mm-thick slice was obtained for the push-out test, yielding 120 specimens (40 teeth × 3 thirds) (Fig. 1).

1

**Figure 2:**
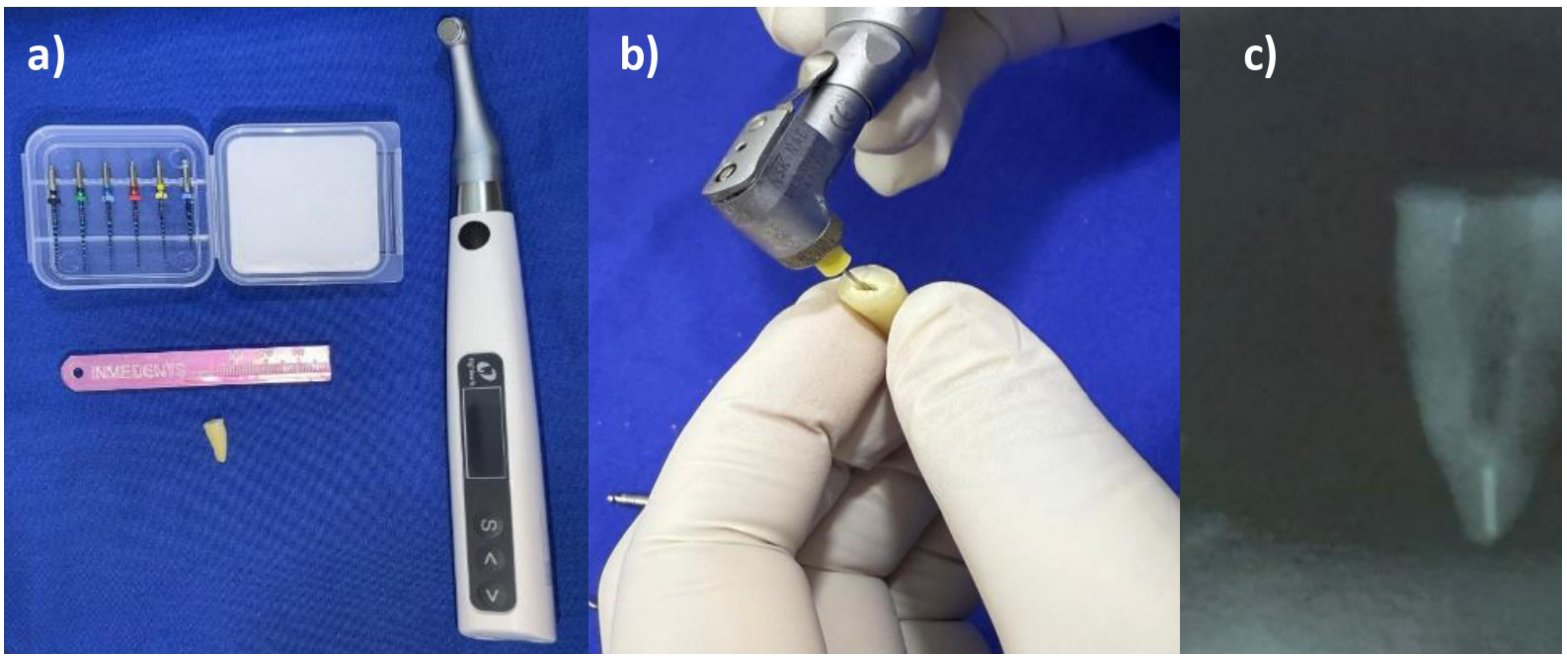
a) Root canal preparation treatment, b) Obturation removal, c) Radiographic evaluation of the spacing for the post.

3. Sample preparation The premolars were gently cleaned with a dentin scraper (HuFriedy, HuFriedy Group, Chicago, IL, USA) to remove soft-tissue debris. The crowns were then sectioned perpendicular to the long axis at the cementoenamel junction using a water-cooled diamond disc (Jota®, Jota AG, Rüthi, Switzerland) mounted on a low-speed micromotor (NSK EX203C, NSK, Tokyo, Japan) (19), obtaining a standardized root length of 15 mm measured from the root apex. All procedures were performed by a single calibrated operator. 4. Root canal treatment Root canal instrumentation was performed using K-type stainless-steel hand files up to size #30 (Mani Inc., Kyoto, Japan) and rotary nickel-titanium instruments (Shenzhen, Guangdong, China). Instrumentation was carried out at a standardized working length of 14 mm using three gentle in-and-out strokes, with light circumferential brushing on withdrawal. After each instrument, the canal was irrigated with 1 mL of 2.5% sodium hypochlorite (Odin Corp., Lima, Peru). The canals were then dried with absorbent paper points (Sure Endo, Seoul, Korea). Root canal obturation was performed by lateral condensation using gutta-percha cones (Spident Co., Incheon, South Korea) and an epoxy resin-based sealer (ViOseal, Spident Co., Incheon, South Korea). Specimens were stored in saline solution at 37 °C until post-space preparation. 5. Root canal preparation To remove the obturation material, a Peeso reamer up to size #3 (Dentsply Maillefer, Ballaigues, Switzerland) was used, followed by a 1.2 mm post-space drill from the manufacturer's post system (Fiber Post AAA, Sadguru Dental, Shenzhen, China), to prepare a post space 11 mm in depth while preserving 3 mm of apical gutta-percha to maintain the apical seal and minimize the gap between the post and the remaining gutta-percha (18). The canal was then refined with the drill corresponding to the glass fiber post system (Fiber Post AAA, Sadguru Dental, Shenzhen, China) to ensure a uniform diameter. Post-space preparation was confirmed radiographically (Fig. 2).

2

**Figure 3:**
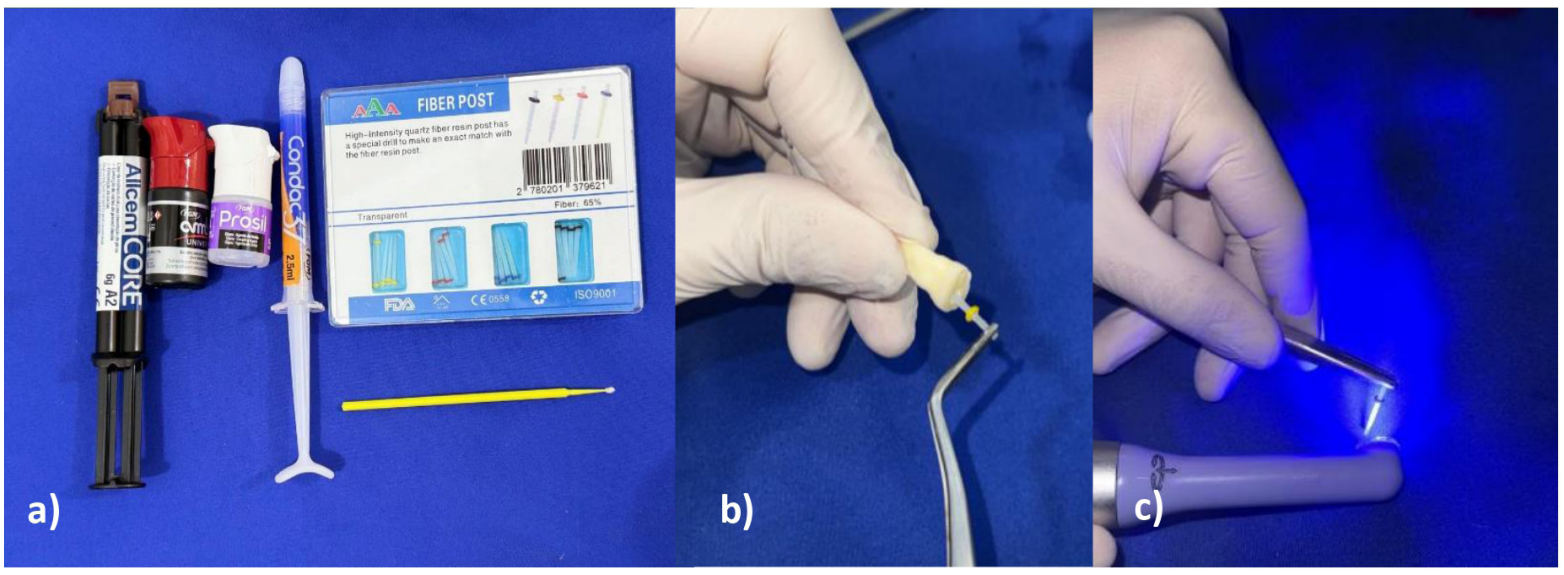
a) Materials used for cementation, b) Cementation of the glass fiber post, c) Light-curing of post.

6. Post surface treatment The teeth were randomly assigned to four experimental groups according to silane solvent evaporation time: Group 1, Control (no silane application); Group 2, 60 s; Group 3, 120 s; and Group 4, 180 s. The post surfaces were cleaned with 70% ethanol (Prolink Indústria Química Ltda., São Paulo, Brazil) (19 , 20), etched with 37% phosphoric acid (Condac, FGM, Joinville, Brazil) for 15 s, and rinsed with water for 60 s (20). All posts received the same cleaning/etching procedure and universal adhesive application; only the silane step (and its solvent evaporation time) was omitted in the control group. In Groups 2-4, the posts were gently air-dried and silanized with Prosil (FGM, Joinville, Brazil), allowing solvent evaporation according to the assigned time (60, 120, or 180 s) (24). Subsequently, a universal adhesive (Ambar, FGM Dental Group, Joinville, Brazil) was applied, air-thinned with a gentle air stream, and light-cured for 10 s (25). In Group 1, no silane was applied; the universal adhesive was applied using the same procedure (air-thinning and 10-s light-curing). The posts were cemented into the root canals using a dual-cure resin cement (Allcem Core, FGM Dental Group, Joinville, Brazil), applied both inside the canal and onto the post. Light-curing was then performed using an LED curing unit (Woodpecker Medical Instrument Co., Ltd., Changsha, China) at an irradiance of 1000 mW/cm² for 40 s. After polymerization, radiographic evaluation of cementation was performed for all specimens (26). The specimens were subsequently stored in a humid environment at 37 °C for 48 h (25) (Fig. 3).

3

7. Push-out Bond Strength Test The roots were sectioned perpendicular to the long axis with a diamond disk connected to a motor (DREMEL® 300 Series, Mt. Prospect, Illinois, USA) under constant cooling to divide each root into three thirds (coronal, middle, and apical) by two perpendicular cuts; from each third, one 1-mm-thick slice was obtained for the push-out test. The slice thickness was checked with a digital caliper (Mitutoyo Sul Americana, Suzano, SP, Brazil), and the specimens were stored for 24 h at 37 °C (19). Each slice was marked on its coronal surface using a permanent marker. The specimens were positioned on a stainless-steel support jig with the coronal side facing the base. The posts were dislodged using cylindrical plungers of different diameters (0.5-1.0 mm), selected according to the diameter of the dentin-cement-post interface. The plunger was aligned with the post axis and positioned to contact only the post, avoiding any contact with the surrounding cement or dentin. Push-out testing was performed using a universal testing machine (CMT-5L, LG, Shandong, China) by applying a compressive load in the apical-to-coronal direction at a crosshead speed of 0.75 mm/min. Bond strength was calculated by dividing the maximum load at failure (N) by the bonded area (MPa = N/mm²) (26). 8. Statistical analysis Data were imported into SPSS v24.0 from a Microsoft Excel 2019 spreadsheet. Descriptive statistics included mean and standard deviation; results were also presented graphically. For inferential analysis, normality (Shapiro-Wilk) and homoscedasticity (Levene) were assessed. For each root third (coronal, middle, and apical), Welch's ANOVA was used for comparisons among the four evaporation-time groups, followed by the Games-Howell post hoc test. Statistical significance was set at p &lt; 0.05. To avoid pseudoreplication, only one specimen per root third per tooth was analyzed. Therefore, statistical comparisons were performed separately for each root third, with each observation corresponding to a different tooth within that third (n = 40 per third; n = 10 per group).

## Results

When comparing the bond strength (MPa) of the silane-treated glass fiber posts at different times, significant differences were observed in the middle root third (p &lt; 0.001). In addition, in the coronal third and apical third, there were no significant differences between the different treatment times and those that were not treated with silane (p = 0.164 and p = 0.196, respectively) (Table 1).

Table 1

**Figure 4:**
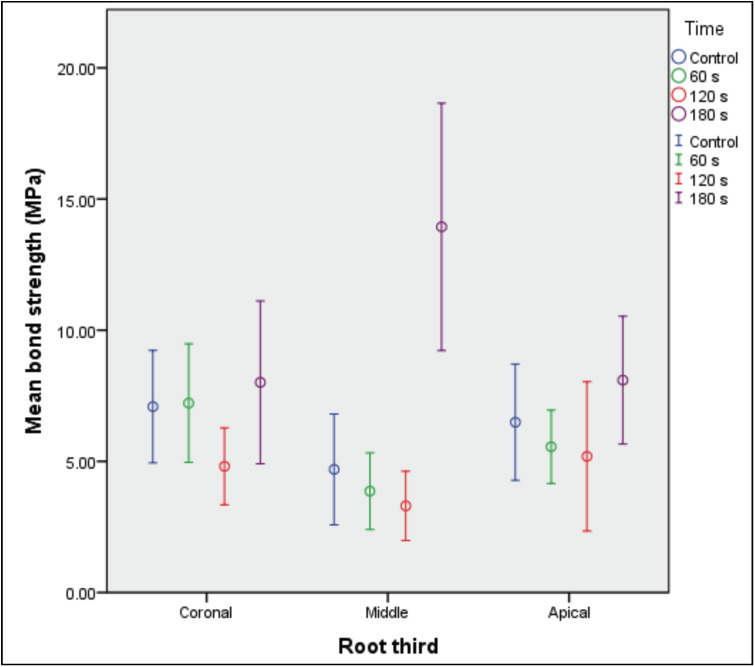
Bond strength averages at 95% confidence interval of silane-treated glass fiber posts at different times, according to root third.

In the middle root third, it was evidenced that posts treated with silane for 180 s had significantly higher bond strength than posts treated for 60 s (p = 0.004), 120 s (p = 0.003), and in those without silane treatment (p = 0.007) (Table 2, Fig. 4).

Table 2

4

When comparing the bond strength (MPa) of the glass fiber posts between root thirds, significant differences were observed in the posts treated with silane for 60 s (p = 0.033). In addition, there were no significant differences in those that were not treated with silane (p = 0.213), and in those that were treated with silane for 120 s (p p = 0.190) and for 180 s (p = 0.062) (Table 3).

Table 3

**Figure 5:**
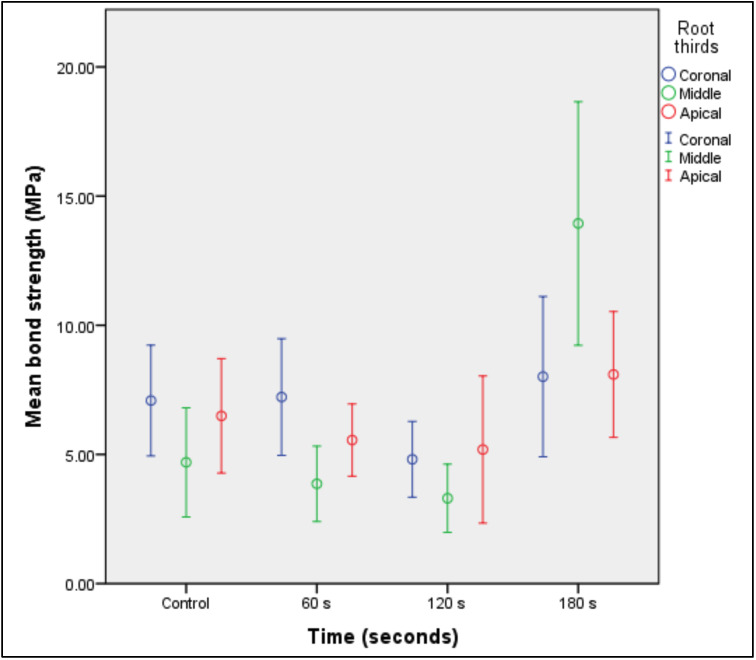
Mean bond strength with 95% confidence intervals among root thirds, according to silane treatment time.

In the posts treated with silane for 60 s, it was evident that the coronal root third had significantly greater bond strength than the middle third (p = 0.032). However, it presented similar bond strength to the apical third (p = 0.357). On the other hand, the middle third did not present significant differences with the apical third (Table 4, Fig. 5).

Table 4

5

## Discussion

The present study evaluated whether differences in solvent evaporation time after silane application (60, 120, and 180 s), compared with a control group without silanization, affect the push-out bond strength of glass fiber posts cemented to root dentin, as assessed by root third (coronal, middle, and apical). This approach was adopted because the drying/evaporation step is critical for promoting a stable silane film and facilitating chemical interaction with the resin matrix (15 , 16). Overall, the findings showed significant differences between protocols only in the middle third, where evaporation for 180 s resulted in higher values compared with 60 s, 120 s, and the group without silane. Accordingly, the null hypothesis was rejected. Solvent evaporation for 180 s increased bond strength in the middle third, whereas the 60 s and 120 s groups and the control group remained at lower values. In contrast, no significant differences were observed between protocols in the coronal or apical thirds. This is in agreement with a study reporting that longer silane solvent evaporation is associated with improved immediate adhesive performance, particularly at 180 s (24). In addition, longer evaporation reduces residual solvent and favors the formation of a more stable and continuous silane layer (siloxane bonds) on the post, thereby enhancing its chemical coupling with the methacrylate-based resin cement during polymerization (15 , 16). Conversely, insufficient evaporation may retain solvent and compromise the quality of the adhesive interface, which is recognized as a critical factor in the performance of silane agents in dentistry (15 , 16). Some studies have not shown consistent benefits of silanization (12). These discrepancies are probably due to variations in the overall protocol, including the type of silane and its application conditions, the pretreatment of the post surface (and its effect on the interaction with cement), and the cementation strategy employed. In this regard, it has been reported that post surface treatments (e.g., conditioning prior to silanization) significantly modify adhesion, and that the effect of silane may depend on such preconditioning (11 , 26). Furthermore, evidence shows that the adhesive gain attributable to silanization is heterogeneous and highly dependent on the post-treatment-cement combination (14). In addition, different systems and types of resin cements are associated with variations in intraradicular dentin bond strength, which could also contribute to discrepancies between studies employing different cements and adhesive strategies (7 - 9 , 13 , 25). When the results were analyzed by root third, the middle third showed lower bond strength than the coronal third at 60 s, whereas at 180 s the middle third exhibited the highest value. This suggests that the effect of silane is not expressed uniformly along the canal, because intraradicular adhesion is influenced by the operative conditions within each third (7 - 10). In the coronal third, better clinical access and more efficient photoactivation facilitate more predictable polymerization and more uniform operative control during cementation, so the additional benefit of optimizing post treatment may be less evident (7 - 9). In the apical third, in contrast, site-specific limitations (restricted access, more complex moisture control, high C-factor geometry, and lower depth polymerization efficiency) tend to dominate bond strength performance and may mask subtle improvements derived from post conditioning alone (7 - 10). Under this framework, the middle third would act as a "sensitive" zone to the state of the cement-post interface: with short drying (60 s) residual solvent could persist and a less stable silanized layer could form, compromising silane chemical coupling and thus the integrity of the adhesive interface (15 , 16). Conversely, with longer evaporation (180 s) the consolidation of a more stable siloxane network and its integration with methacrylic matrices is favored, allowing the improvement of the cement-post interface to translate into a measurable increase in push-out (9 , 10 , 14 - 16). Therefore, the effect of the protocol was mainly expressed in the middle third with 180 s, while in coronal and apical the differences between times were not evident (8 , 9). From a clinical perspective, this study provides evidence on a specific and easily modifiable operative parameter of the silanization protocol, since prolonging solvent evaporation up to 180 s could improve the quality of the cement-post interface. This finding is relevant because the effectiveness of silane depends not only on its application, but also on conditions that determine the formation of a stable film, including the drying/evaporation stage (15 , 16). Regarding study limitations, it should be considered that the study was in vitro and bond strength was evaluated under controlled and short-term conditions, so extrapolation to clinical performance should be taken with caution (19). Furthermore, the results may be conditioned by the specific combination of post, silane, adhesive/cement, and the overall protocol, since the benefit of silanization has shown variability between systems and methodologies (14). In addition, intraradicular behavior depends on multiple determinants that exceed the surface treatment of the post (8 , 9). Finally, the exclusive use of the push-out assay limits interpretation, as it does not physically and completely reproduce clinical environmental and biomechanical demands (19). Future studies should extend comparison to other adhesive systems and cements, considering that their choice may modify bond strength (20 , 25). In turn, it is recommended to explore alternative silanization formulations and strategies, given that their performance depends on both the chemistry of the agent and the application protocol. Likewise, it is suggested to incorporate hydrothermal aging and functional loading/fatigue, under a standardized methodology, to more robustly evaluate the effect of silane evaporation times as pretreatment of the post. In parallel, it would be pertinent to propose clinical trials that evaluate clinically relevant outcomes (e.g., retention/decementation loss and restorative survival) under well-defined cementation protocols, consistent with the available clinical follow-up evidence on post restorations.

## Conclusions

Prolonged silane solvent evaporation for 180 s improved the bond strength of glass fiber posts in the middle root third compared with shorter times and the control group without silanization. In the coronal and apical thirds, varying the evaporation time did not significantly affect bond strength. However, in the 60 s group, the middle third showed lower bond strength than the coronal third. Taken together, these findings support using 180 s of solvent evaporation after silane application when seeking to maximize bonding in the middle third, whereas prolonging the evaporation time in the coronal and apical thirds does not provide consistent benefits.

## Figures and Tables

**Table 1:** Descriptive values and comparison of the bond strength (MPa) of glass fiber posts treated with silane at different times, according to root third.

Root third	Time	n	Mean	SD	SE	95% CI		p***	p**	p*
						LL	UL			
Coronal	Control (no silane)	10	7.09	3.00	0.95	4.94	9.23	0.062	0.084	0.164
	60 s	10	7.22	3.16	1.00	4.96	9.48	0.503		
	120 s	10	4.81	2.05	0.65	3.35	6.28	0.274		
	180 s	10	8.01	4.34	1.37	4.91	11.11	0.468		
Middle	Control (no silane)	10	4.69	2.95	0.93	2.58	6.81	0.150	0.001	<0.001*
	60 s	10	3.86	2.04	0.65	2.40	5.32	0.832		
	120 s	10	3.30	1.85	0.58	1.98	4.63	0.142		
	180 s	10	13.94	6.59	2.08	9.23	18.66	0.681		
Apical	Control (no silane)	10	6.49	3.09	0.98	4.28	8.71	0.280	0.241	0.196
	60 s	10	5.56	1.96	0.62	4.15	6.96	0.264		
	120 s	10	5.19	3.98	1.26	2.34	8.04	0.208		
	180 s	10	8.10	3.40	1.08	5.66	10.53	0.915		

n: sample size, SD: Standard deviation, SE: Standard error of the mean, 95% CI: 95% confidence interval, LL: Lower limit, UL: Upper limit; ***Based on Shapiro Wilk’s normality test (p>0.05, normal distribution); **Based on Levene’s homoscedasticity test (p>0.05, homogeneous variances); *Based on Welch’s robust ANOVA for one intergroup factor (*p < 0.05, significant differences).

**Table 2:** Multiple comparisons of the bond strength (MPa) of silane-treated glass fiber posts at different times in the middle third of the root.

Root third	Time	Time		
		60 s	120 s	180 s
Middle	Control (no silane)	p = 0.883	p = 0.599	p = 0.007*
	60 s		p = 0.917	p = 0.004*
	120 s			p = 0.003*

* Based on Games–Howell post hoc (p < 0.05, significant differences)

**Table 3:** Comparison of the bond strength (MPa) of glass fiber posts between root thirds, according to the silane treatment time.

Time	Root third	n	Mean	SD	SE	95% CI		p***	p**	p*
						LL	UL			
Control (no silane)	Coronal	10	7.09	3.00	0.95	4.94	9.23	0.062	0.764	0.213
	Middle	10	4.69	2.95	0.93	2.58	6.81	0.150		
	Apical	10	6.49	3.09	0.98	4.28	8.71	0.280		
60 s	Coronal	10	7.22	3.16	1.00	4.96	9.48	0.503	0.092	0.033*
	Middle	10	3.86	2.04	0.65	2.40	5.32	0.832		
	Apical	10	5.56	1.96	0.62	4.15	6.96	0.264		
120 s	Coronal	10	4.81	2.05	0.65	3.35	6.28	0.274	0.006	0.190
	Middle	10	3.30	1.85	0.58	1.98	4.63	0.142		
	Apical	10	5.19	3.98	1.26	2.34	8.04	0.208		
180 s	Coronal	10	8.01	4.34	1.37	4.91	11.11	0.468	0.116	0.062
	Middle	10	13.94	6.59	2.08	9.23	18.66	0.681		
	Apical	10	8.10	3.40	1.08	5.66	10.53	0.915		

n: sample size, SD: Standard deviation, SE: Standard error of the mean, 95% CI: 95% confidence interval, LL: Lower limit, UL: Upper limit; ***Based on Shapiro Wilk’s normality test (p>0.05, normal distribution); **Based on Levene’s homoscedasticity test (p>0.05, homogeneous variances).05, normal distribution); **Based on Levene’s homoscedasticity test (p>0.05, homogeneous variances); *Based on intergroup one-factor ANOVA test (*p < 0.05, significant differences).

**Table 4:** Multiple comparisons of the bond strength (MPa) of glass fiber posts between root thirds, with 60 seconds of silane treatment.

Time	Root third	Root third	
		Middle	Apical
60 s	Coronal	p = 0.032*	p = 0.357
	Middle		p = 0.170

* Based on Games–Howell post hoc (p < 0.05, significant differences).

## Data Availability

The data supporting the findings of this study are available from the corresponding author upon reasonable request.
